# Assessment of bioaerosol particle characteristics at different hospital wards and operating theaters: A case study in Tehran

**DOI:** 10.1016/j.mex.2018.11.021

**Published:** 2018-12-01

**Authors:** Fatemeh Bolookat, Mohammad Sadegh Hassanvand, Sasan Faridi, Mostafa Hadei, Masoumeh Rahmatinia, Mahmood Alimohammadi

**Affiliations:** a Department of Environment Engineering, Faculty of Environment & Energy, Islamic Azad University Science and Research Branch, Tehran, Iran; b Center for Air Pollution Research, Institute for Environmental Research, Tehran University of Medical Sciences, Tehran, Iran; c Department of Environmental Health Engineering, School of Public Health, Tehran University of Medical Sciences, Tehran, Iran; d Student Research Committee, Department of Environmental Health Engineering, School of Public Health and safety, Shahid Beheshti University of Medical Sciences, Tehran, Iran; e Center for Water Quality Research (CWQR), Institute for Environmental Research (IER), Tehran University of Medical Sciences, Tehran, Iran; g Health Equity Research Center (HERC), Tehran University of Medical Sciences, Tehran, Iran

**Keywords:** Bacterial and fungal samples were collected using the passive sampling method of 1/1/1 scheme during a six months' period in the specialty and subspecialty the hospital from August 2015 to February 2016, Hospital airborne bioaerosols, Indoor air, Fungal bioaerosol, Tehran

## Abstract

This study was aimed to investigate the types and number of bacterial and fungal bioaerosols in indoor air of hospitals according to the type of wards and operating theaters. Bacterial and fungal samples were collected using the passive sampling method of 1/1/1 scheme during a six months' period in the Khatam-Al-Anbia hospital, Tehran, Iran. A simple linear regression was used to determine the relationship between bioaerosol concentrations and the number of active beds. Bacterial bioaerosol concentrations were mainly higher than fungi in all sampling sites. A significant association was found between airborne fungal concentrations and the numbers of beds (R^2^ = 0.76, p < 0.05), but not observed for bacteria (R^2^ = 0.02, p < 0.05). Our findings provided an exposure database of airborne bacterial and fungal bioaerosol in hospital wards and operating theaters in Tehran.

• Due to the importance of the exposure risk to bioaerosols for patients and medical personnel, we focused on identification of the density and diversity of bacterial and fungal bioaerosols in different wards and operating theaters.
• Our results showed that the numbers of the beds have a significant effect on airborne fungal concentrations.
• The results of this study can be used to set indoor air quality standards for hospital wards and operating theatres.

## Graphical abstract

**Figure:**
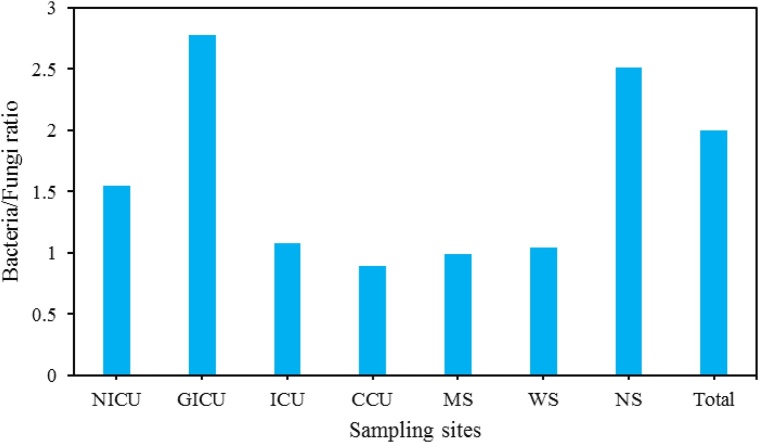

**Specifications Table**

**Table:** 

Subject area	Environmental health; Airborne pollutants
More specific subject area	Bioaerosol
Method name	Bacterial and fungal samples were collected using the passive sampling method of 1/1/1 scheme during a six months' period in the specialty and subspecialty the hospital from August 2015 to February 2016.
Name and reference of original method	Doi: org/https://doi.org/10.1016/j.jhin.2016.03.017
Resource availability	Data can be found within this article

## Method details

Exposure to bioaerosols, as an important class of particulate matter (PM), have become one of the most issues in indoor air quality (IAQ) topics for occupational and public health [[1], [2], [3], [4]]. In particular, hospitals have a more complex and different environment rather than other occupational or residential ones. This is due to continuous working cycle, and special types of air pollutants, including pathogenic and chemical or biological agents [5]. Bioaerosols can originate from humans (including patients), various indoor hospital characteristics, and outdoor environmental sources [2]. It has been found that about 5–34% of indoor air pollution in different indoor environments such as hospitals, dentist ofﬁces, shopping centers, subway systems, public libraries, and other workplaces can be attributable to bioaerosols, particularly bacterial and fungal bioaerosols [3,6]. Bioaerosols are compounds with biological origin, including dead or live bacterial and fungal spores, bacterial peptidoglycans and endotoxins, fungal mycotoxins and β (1, 3)-glucans, viruses, pollen grains, airborne algae, plant debris, and fragments of microbial insects, fur ﬁbres and skin fragments from animals and humans [[7], [8], [9]]. Among this wide range of species, bacterial and fungal aerosols have been introduced as the most important bioaerosols [3]. Bioaerosols in general, have been associated to several adverse health effects. Considering the significance of this issue, the World Health Organization (WHO) published a guideline on IAQ, in 2009 [10]. Well-documented environmental health studies demonstrate that exposure to bioaerosols, exceeding maximum acceptable levels can lead to non-infectious diseases, infectious diseases, including nosocomial infections or healthcare associated infections, acute toxic effects, cancer and even death, especially for immunosuppressed persons [1,[11], [12], [13], [14], [15], [16], [17], [18]]. Nosocomial infections cause significant health and economic problems [19,20]. About 1.4 million people are suffering from such infections at any one time worldwide [21]. The consequences of these infections may include extended hospital stay, long-term disability, increased resistance to antibiotics, a huge financial burden for government and patients, and excess deaths [22]. Several factors can affect the number and types of bioaerosols in hospital environments. The density and diversity of biological contaminants in hospitals depend on various factors such as the type of ward, the number and activity of visitors and patients, hospital room design, disinfection, ventilation, temperature and humidity, etc. [23,24]. Accordingly, assessment of bioaerosols in hospital environments, particularly in high risk operating theatres where patients are more susceptible is highly essential [25]. Despite all these studies, many issues remain debating. First, identifying the most high-risk wards, and the dominant species of microorganisms are always critical. Second, different site-specific factors can affect the type and number of bioaerosols. The effect of number of active beds is a less-documented factor. Third, antimicrobial resistance (AMR) can be detected by these studies [22,26]; since, the presence of settle-able bioaerosols can be an indicator for effectiveness of disinfection processes in hospitals. Therefore, it is never repetitive to study bioaerosols in hospitals quantitatively and qualitatively. Regarding these issues, this study was perform to investigate the density and diversity of bacterial and fungal bioaerosols in different wards and operating theaters of a high crowded hospital (Khatam-ol-Anbia) in Tehran, Iran. In addition, the effect of number of active beds on bioaerosols concentrations were investigated.

## Methods

### Sampling sites

This cross-sectional study was conducted at different wards of specialty and subspecialty Khatam-Al-Anbia hospital, located on Tehran from August 2015 to February 2016. All sampling sites were first categorized into 2 groups, which are hospital wards including CCU (coronary care unit), GICU(general intensive care unit), ICU(intensive care unit), and NICU(neonatal intensive care unit) and operating theaters including MS(men surgery), NS(neonatal surgery), and WS(women surgery). Then measurements of bacterial and fungal bioaerosol concentrations were conducted in all sites including CCU, GICU, ICU, NICU, MS, NS and WS.

### Sampling procedure

Passive sampling provides a valid risk assessment as it measures the harmful part of the airborne population which falls onto critical surfaces [27]. It is the most readily available, economic, and unobtrusive method of bioaerosol sampling, and is based on particles settling by means of gravity, on a collection substrate housed in a settle plate. The concentration is usually expressed as the number of colony-forming units (CFU) within the area of the settling plates for the duration of a specified time-period (for example in units of CFU/m^2^/h) [28]. This is also known as the index of microbial air contamination (IMA) [29]. Passive method captures only the fractions of bacteria and fungi that can sediment in a specific period of time. This fraction can be very critical in hospital environments, because bacteria and fungi can contaminate different surfaces and equipment such as surgical cut in operating theatres [29,30]. To determine the Index of Microbial Air Contamination (IMA) in hospital wards and operating theaters, passive sampling was performed [25,[31], [32], [33]], because there was no ofﬁcial permission to use any active sampling equipment. The sampling was performed from 10:00 AM to 16:00 PM, during a six months' period. 90-mm petri dishes containing Tryptic cycloheximide soy agar and Sabouraud chloramphenicol dextrose agar were used for bacterial and fungal samples, respectively [24,[34], [35], [36]] The 1/1/1 scheme as a standardized method was used to collect bioaerosols [28]. The plates containing the sterile culture medium were located for sampling at the height of 1 m above the ground level and with a distance more than 1 m from the walls, doors, windows and barriers for 1 h. At the end of the sampling, the petri dishes were immediately transferred to the laboratory and incubated at 37 ± 1 ℃ for 2 days for bacteria [37,38] and at 25 ± 1 ℃ for 4 days for fungi [[39], [40], [41]]. 36 samples were taken from each site, including 18 samples for fungal, and 18 samples for bacterial analyses. In total, 252 samples (126 bacterial samples and 126 fungal samples) were collected for bacterial and fungal bioaerosols. Bacterial and fungal samplings were simultaneously conducted in all sampling sites. In addition, the numbers of the patient beds in all sampling sites was recorded by observation in each sampling day to assess the association between the numbers of the beds and the concentrations of bacterial, fungal and total bioaerosols.

### Identification of bacterial and fungal bioaerosols

After adding 500 mg/L of cycloheximide to inhibit the proliferation of fungi, bacterial samples were incubated at 37 °C for at 24–48 h. Genera of bacteria was identified according to Bergey's manual. After Gram staining, a biochemical test was carries out to identify genera using an automatic identification system – VITEK (Model VITEK 32 system, bioMerieux Inc., France) [36,42]. After adding 100 mg/L of chloramphenicol to inhibit the proliferation of bacteria, fungal samples were incubated at 25 °C for 72 h. An optical microscope with magnification of 400× was used to assess the shape and color of colonies and spores, vegetative hyphae, and sexual and asexual reproductive organs. The identification of genera was accomplished based on the taxonomic method of Ainsworth and Baron [2,34]. Finally, the results were expressed in colony forming units per square meter per hour (CFU/m^2^/h) [10,25].

### Data analysis

Descriptive statistics were calculated to describe the concentration of bacterial and fungal aerosols. To evaluate differences between bacterial, fungal and total bioaerosols (sum of the bacterial and fungal bioaerosols) in different sampling sites, one-way ANOVA test was employed. In addition, a simple linear regression model was used to evaluate the association between the numbers of beds and bioaerosol concentrations. All the statistical elaborations were conducted using Minitab software version 17. P-values less than 0.05 were considered as statistically signiﬁcant in all analyses.

## Results and discussion

### Concentration of bacterial and fungal bioaerosols

A summary of statistics for the bacterial and fungal bioaerosol (CFU m^–2^ h^−1^) concentrations in the sampling sites are given in Table 1. A total of 746 and 875 CFU of fungal and bacterial bioaerosol from 252 plates (126 SDA plates and 126 TSA plates) were collected by passive sedimentation technique in all the sampling sites. As can be seen in Table 1, the bacterial, fungal and total bioaerosol levels varied on a large scale within the sampling sites. According to Table 1, bacterial and fungal concentrations varied from 127 to 1783 and 127 to 1529 CFU m^–2^ h^−1^ in all sampling sites, with maximum mean values for GICU and CCU, respectively. The average of fungal concentrations for all sampling sites were 719 (NICU), 622 (GICU), 899 (ICU), 1191 (CCU), 884 (MS), 804 (WS), and 527 (NS) CFU m^–2^ h^−1^; while, the average of bacterial concentrations was 963, 1097, 920, 1012, 849, 715, and 884 CFU m^–2^ h^−1^, respectively. Based on the average concentrations, the total fungal bioaerosols were higher than the bacterial bioaerosols in the CCU, MS, and WS, whereas total bacterial bioaerosols were higher than the fungal bioaerosols observed in the NICU, GICU, ICU, and NS. There was a considerable statistic difference in the concentration of fungal bioaerosols in the hospital wards and operating theaters (p < 0.05). The same difference was found for bacterial bioaerosols between hospital wards and operating theaters (p < 0.05). In overall, the average of total bacterial concentration (919 CFU m^–2^ h^−1^) was significantly higher than fungal bioaerosol (796 CFU m^–2^ h^−1^) in current study (Table 1). Our results are consistent with those of [2,10,34,36,40,[43], [44], [45]], who showed that bacterial bioaerosol concentrations were higher than fungal species. This finding can be attributed to the larger number of bacterial bioaerosol than fungal species in natural resources, such as the soil [46], and the air over vegetated regions [47]. On the other hand, the higher sedimentation of fungal bioaerosols due to their larger aerodynamic diameter (1–30 μm) compared with bacterial bioaerosols (< 2.5 μm) can change the contribution of settled bacterial and fungal species to the total burden of microbial species [8,10].

**Table 1:** Descriptive statistics of the bacterial and fungal bioaerosol (CFU m^–2^ h^−1^) concentrations in the Sampling sites.

Sampling sites	Fungal aerosols (n = 126, (7 × 18))			Bacterial aerosols (n = 126, (7 × 18))		
	Min-Max	Mean ± SD	Median	Min-Max	Mean ± SD	Median
NICU	382–1147	719 ± 251	637	255–1529	963 ± 328	1019
GICU	127–1019	622 ± 266	637	510–1529	1097 ± 303	1019
ICU	510–1147	899 ± 183	892	510–1529	920 ± 285	892
CCU	764–1529	1191 ± 246	1274	637–1401	1012 ± 241	1019
MS	637–1274	884 ± 188	892	382–1147	849 ± 231	892
WS	382–1147	804 ± 162	892	255–1019	715 ± 215	764
NS	127–1019	527 ± 240	510	127–1783	884 ± 396	892
Total	127–1529	796 ± 302	764	127–1783	919 ± 305	892

**Fig. 1:**
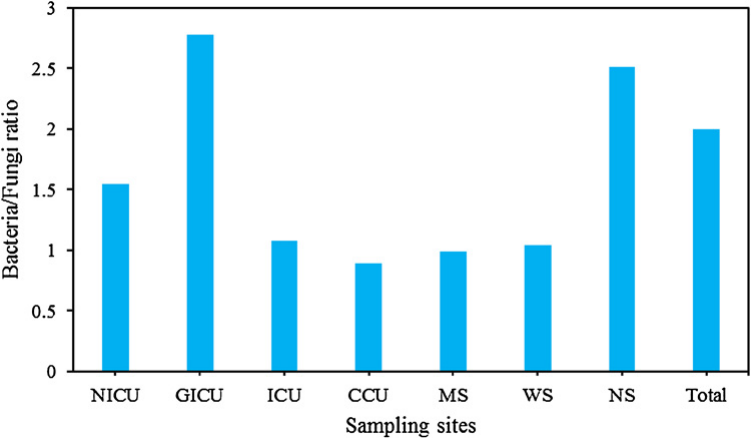
Bacterial /fungal bioaerosol concentration ratios in all sampling sites during the study.

Fig. 1 shows the ratios of bacterial/fungal (B/F) bioaerosol in all the sampling sites. The mean value of bacterial/fungal (B/F) bioaerosol ratio during the study ranged from 0.89 to 2.78, with a value of 1.55, 2.78, 1.08, 0.89, 0.99, 1.04, and 2.51 for NICU, GICU, ICU, CCU, MS, WS, and NS, respectively. Therefore, the B/F ratio in NICU, GICU, ICU, WS, NS were above 1.0. Likewise, the highest B/F bioaerosol concentrations ratio observed in GICU (2.78) and the lowest values recorded in CCU (0.89). Our findings indicated that the mean total bacteria/total fungi concentration ratio during the study was 2 (Fig. 1).

### Frequency and types of species

**Fig. 2:**
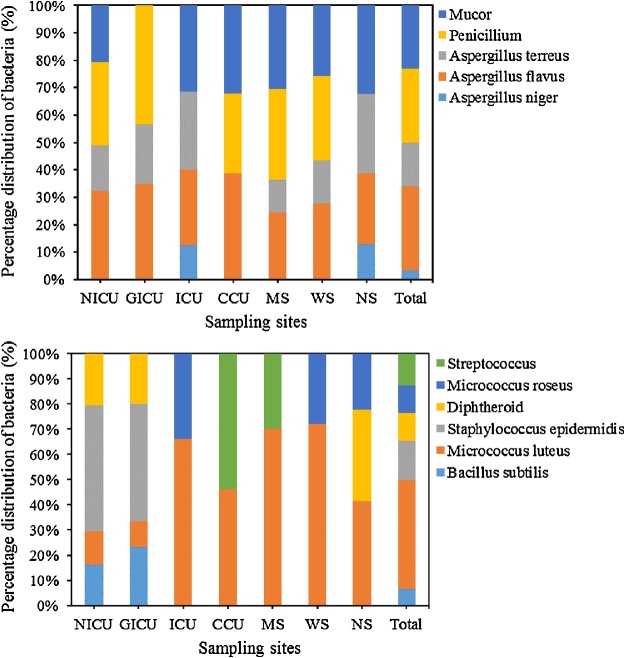
Percentage of fungal (a) and bacterial (b) bioaerosols in the sampling sites.

The percentage of fungal (a) and bacterial (b) bioaerosols in the sampling sites is shown in Fig. 2. Our findings indicated that *Penicillium* spp., *Aspergillus* spp., and *Mucor spp*. (Fig. 2a) were the most observed mold genera in all sampling sites. Most studies support our results and indicate the prevalence and persistence of some fungal species in the different hospital environments [2,6,25,33,36,48]. The identiﬁed Aspergillus fungal bioaerosols in hospital wards and operating theaters belonged to *A. terreus*, *A. flavus*, and *A. niger* (Fig. 2a). As shown in Fig. 2a, NICU, MS, and WS had similar fungal bioaerosol species/genera, including *Mucor* spp., *Penicillium* spp., *Aspergillus terreus*, and *Aspergillus flavus*. In addition, *Mucor* spp., *Aspergillus terreus*, *Aspergillus flavus*, and *Aspergillus niger* were found as dominant fungal bioaerosol species in NS and ICU. In overall, *Aspergillus* spp. (49.9%) were the most dominant fungal genera, followed by *Penicillium* spp. (26.9%), and *Mucor* spp. (23.2%). This finding could be result by the fact that several genus of Aspergillus have xerophilic property which might enable them to survive in the hospital wards and operating theaters for relatively long time than other fungal bioaerosols [36]. Several studies reported that Aspergillus species in hospital air leads to nosocomial infections [2,49,50]. The most isolated bacterial bioaerosols can be ranked as following: *Micrococcus luteus* (43.4%) > *Staphylococcus epidermidis* (15.5%) > *Streptococcus* spp. (12.5%) > *Diphtheroid* spp. (11.3%) > *Micrococcus roseus* (10.8%) > *Bacillus subtilis* (6.4%) in the all samples. Our ﬁndings are in good agreement with the other studies [6,10,36,2,43,45,[51], [52], [53]]. The most prevalent bacterial bioaerosols were identified to be *Micrococcus luteus* with 41.5%, 72.3%, 70.0%, 66.2% of total bacterial bioaerosols in NS, WS, MS, and ICU, respectively; and *Staphylococcus epidermidis* with 46.6% and 50.0% of total bacterial bioaerosols in GICU and NICU, respectively. Predominant bacterial bioaerosols in CCU were *Streptococcus* spp. (53.8%), and *Micrococcus luteus* (46.2%), respectively. All the bacterial bioaerosols in our study were gram-positive. The ratio of gram-positive to total bacteria has been previously reported to be 88% [2], 89–100% [54], 90–92% [53], 89% [51], 100% [8,10], 92–100% [55], and 77.6–80.8% [9]. Gram-positive bacteria are present in many macro- and micro-environments and are the normal flora of the skin, mucous membranes, hair of human beings and animals [9,34,56,57]. In addition, the resistance of gram-positive species is much more than gram-negative species, and can survive even under unfavorable ambient and enclosed environmental conditions [24,41,42].

### Number of beds

**Fig. 3:**
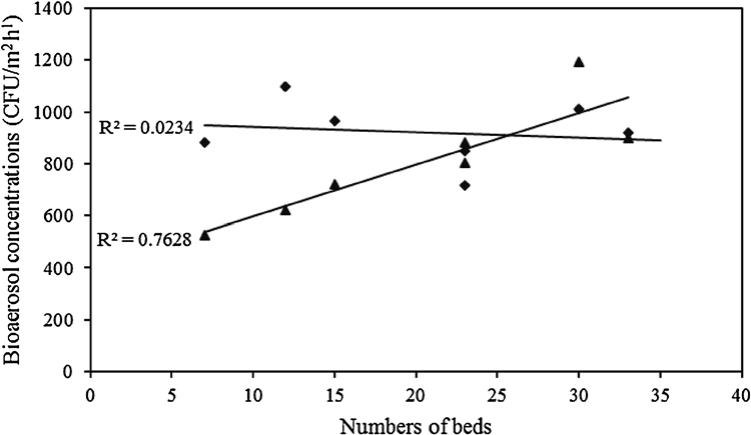
Association between the numbers of beds and fungal bioaerosol (FB) and bacterial bioaerosol (BB) concentrations in all the sampling sites.

We found the highest fungal bioaerosol concentrations in CCU. This result can be attributed to the larger numbers of the beds in CCU. As shown in Fig. 3, linear regression analysis revealed a significant strong association between fungal bioaerosols and the numbers of beds (R^2^ = 0.76, p < 0.05) [44,50]. On the other hand, a poor association was found between the concentrations of bacterial bioaerosols and the numbers of beds (R^2^ = 0.02, p < 0.05), indicating that bacterial bioaerosols concentrations in the sampling sites have been affected from other parameters other than the number of beds. These factors may include the number of health-care workers, visitors and patients' coughing and sneezing [2,44,58,59].

### Comparison to available standards

**Fig. 4:**
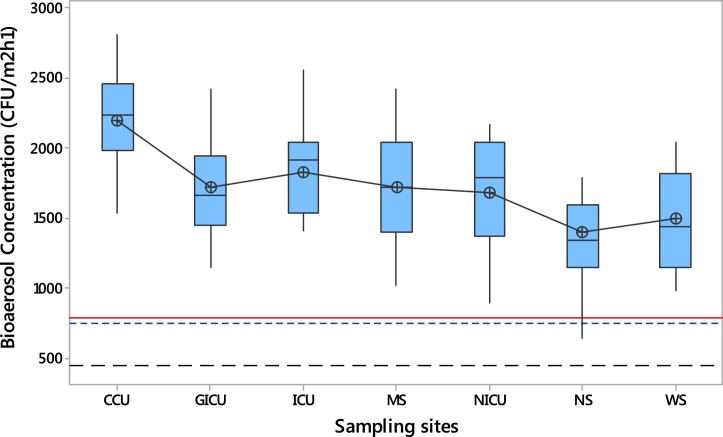
Boxplot for bioaerosol concentrations during the study period in the sampling sites compared to the available standards (maximum levels of the index of microbial air contamination: MAL of IMA) in operating theatres (red line), the optimal (black long dash line) and acceptable levels (blue dash line) of the index of microbial air contamination (OL of IMA and AL of IMA) in hospital wards.

Fig. 4 shows the boxplots of the total bioaerosol concentrations in the sampling sites compared to the available standards. As illustrated in Fig. 4, total bioaerosol concentrations indicated a statistically noticeable difference (p < 0.05) in all sampling sites, especially between CCU and NS. Since no national guidelines or standard limits for the index of microbial air contamination are provided by Iranian official documents, we considered the Swiss Hospital Association standards and other standards available as maximum levels of the index of microbial air contamination (MAL of IMA) in operating theatres (red line, ≤ 786 CFU m^–2^ h^−1^ or ≤ 5 CFU/90 mm diameter plate/h), the optimal (black long dash line, ≤ 450 CFU m^–2^ h^−1^) and acceptable levels (blue dash line, ≤ 750 CFU m^–2^ h^−1^) of the index of microbial air contamination (OL of IMA and AL of IMA) in hospital wards. As illustrated in Fig. 4, in both the operating theatres (MS, WS, and NS) and the hospital wards (GICU, CCU, ICU, and NICU), the lower quartile of total bioaerosol concentrations exceeded the MAL of IMA, the OL of IMA and AL of IMA. Based on the mean total bioaerosol concentrations, the highest mean total bioaerosol values was detected in the CCU (2194 CFU m^–2^ h^−1^), followed by the ICU (1819 CFU m^–2^ h^−1^), MS (1719 CFU m^–2^ h^−1^), GICU (1716 CFU m^–2^ h^−1^), NICU (1673 CFU m^–2^ h^−1^), WS (1494 CFU m^–2^ h^−1^) and NS (1393 CFU m^–2^ h^−1^) rooms (Fig. 4). In addition, as shown in Fig. 4 the mean total bioaerosol concentrations in the CCU, ICU, GICU, and NICU wards were 2.93, 2.43, 2.29, and 2.23 times higher than the AL of IMA, respectively; whereas, 4.84, 4.04, 3.81, and 3.71 times higher than the OL of IMA. In case of operating theaters, the mean total bioaerosol concentrations in the MS, WS, and NS were 2.19, 1.90, and 1.77 times higher than the MAL of IMA, respectively. Thus, we could conclude that the investigated indoor hospital environments were heavily contaminated with airborne bacterial and fungal bioaerosols at a high level. Therefore, patients, staff, and other subjects were heavily exposed to total bioaerosol concentrations exceeded the standards available in the whole sampling sites.

## Conclusion

The present study was carried out to investigate the compositions and the concentrations of airborne bacterial and fungal bioaerosols in hospital wards and operating theaters in Tehran. Bacterial and fungal bioaerosols were isolated from all the samples. Our results showed that concentrations of bacterial, fungal and total bioaerosols at these sites were highly variable, especially for fungal bioaerosols. In addition, our findings suggested that the numbers of the beds have a significant positive and strong effect on airborne fungal concentrations. Despite their signiﬁcant impact on human health, hospital airborne bioaerosols and their affecting factors are not well understood. Comprehensive studies are necessary to determine the role of other factors including the number of health-care workers and visitors, coughing, sneezing, etc. on the concentrations of airborne bioaerosols. The results of this study can be used to set indoor air quality standards for hospital wards and operating theatres, especially in case of maximum allowable number of beds.

## Conflict of interest

The authors declare that they have no conflict of interest.
